# Excess Deaths Associated with COVID-19, by Age and Race and Ethnicity — United States, January 26–October 3, 2020

**DOI:** 10.15585/mmwr.mm6942e2

**Published:** 2020-10-23

**Authors:** Lauren M. Rossen, Amy M. Branum, Farida B. Ahmad, Paul Sutton, Robert N. Anderson

**Affiliations:** 1 National Center for Health Statistics, CDC.

As of October 15, 216,025 deaths from coronavirus disease 2019 (COVID-19) have been reported in the United States*; however, this number might underestimate the total impact of the pandemic on mortality. Measures of excess deaths have been used to estimate the impact of public health pandemics or disasters, particularly when there are questions about underascertainment of deaths directly attributable to a given event or cause (*1*–*6*).^†^ Excess deaths are defined as the number of persons who have died from all causes, in excess of the expected number of deaths for a given place and time. This report describes trends and demographic patterns in excess deaths during January 26–October 3, 2020. Expected numbers of deaths were estimated using overdispersed Poisson regression models with spline terms to account for seasonal patterns, using provisional mortality data from CDC’s National Vital Statistics System (NVSS) (*7*). Weekly numbers of deaths by age group and race/ethnicity were assessed to examine the difference between the weekly number of deaths occurring in 2020 and the average number occurring in the same week during 2015–2019 and the percentage change in 2020. Overall, an estimated 299,028 excess deaths have occurred in the United States from late January through October 3, 2020, with two thirds of these attributed to COVID-19. The largest percentage increases were seen among adults aged 25–44 years and among Hispanic or Latino (Hispanic) persons. These results provide information about the degree to which COVID-19 deaths might be underascertained and inform efforts to prevent mortality directly or indirectly associated with the COVID-19 pandemic, such as efforts to minimize disruptions to health care.

Estimates of excess deaths can provide a comprehensive account of mortality related to the COVID-19 pandemic, including deaths that are directly or indirectly attributable to COVID-19. Estimates of the numbers of deaths directly attributable to COVID-19 might be limited by factors such as the availability and use of diagnostic testing (including postmortem testing) and the accurate and complete reporting of cause of death information on the death certificate. Excess death analyses are not subject to these limitations because they examine historical trends in all-cause mortality to determine the degree to which observed numbers of deaths differ from historical norms. In April 2020, CDC’s National Center for Health Statistics (NCHS) began publishing data on excess deaths associated with the COVID-19 pandemic (*7*,*8*). This report describes trends and demographic patterns in the number of excess deaths occurring in the United States from January 26, 2020, through October 3, 2020, and differences by age and race/ethnicity using provisional mortality data from the NVSS.^§^

Excess deaths are typically defined as the number of persons who have died from all causes, in excess of the expected number of deaths for a given place and time. A detailed description of the methodology for estimating excess deaths has been described previously (*7*). Briefly, expected numbers of deaths are estimated using overdispersed Poisson regression models with spline terms to account for seasonal patterns. The average expected number, as well as the upper bound of the 95% prediction interval (the range of values likely to contain the value of a single new observation), are used as thresholds to determine the number of excess deaths (i.e., observed numbers above each threshold) and percentage excess (excess deaths divided by average expected number of deaths). Estimates described here refer to the number or percentage above the average; estimates above the upper bound threshold have been published elsewhere (*7*). Observed numbers of deaths are weighted to account for incomplete reporting by jurisdictions (50 states and the District of Columbia [DC]) in the most recent weeks, where the weights were estimated based on completeness of provisional data in the past year (*7*).

Weekly NVSS data on excess deaths occurring from January 26 (the week ending February 1), 2020, through October 3, 2020, were used to quantify the number of excess deaths and the percentage excess for deaths from all causes and deaths from all causes excluding COVID-19.^¶^ Deaths attributed to COVID-19 have the *International Classification of Diseases, Tenth Revision* code U07.1 as an underlying or contributing cause of death.

Weekly numbers of deaths by age group (0–24, 25–44, 45–64, 65–74, 75–84, and ≥85 years) and race/ethnicity (Hispanic or Latino [Hispanic], non-Hispanic White [White], non-Hispanic Black or African American [Black], non-Hispanic Asian [Asian], non-Hispanic American Indian or Alaska Native [AI/AN], and other/unknown race/ethnicity, which included non-Hispanic Native Hawaiian or other Pacific Islander, non-Hispanic multiracial, and unknown) were used to examine the difference between the weekly number of deaths occurring in 2020 and the average number occurring in the same week during 2015–2019. These values were used to calculate an average percentage change in 2020 (i.e., above or below average compared with past years), over the period of analysis, by age group and race and Hispanic ethnicity. NVSS data in this report include all deaths occurring in the 50 states and DC and are not limited to U.S. residents. Approximately 0.2% of decedents overall are foreign residents. R statistical software (version 3.5.0; The R Foundation) was used to conduct all analyses.

From January 26, 2020, through October 3, 2020, an estimated 299,028 more persons than expected have died in the United States.** Excess deaths reached their highest points to date during the weeks ending April 11 (40.4% excess) and August 8, 2020 (23.5% excess) (Figure 1). Two thirds of excess deaths during the analysis period (66.2%; 198,081) were attributed to COVID-19 and the remaining third to other causes^††^ (Figure 1).

**FIGURE 1:**
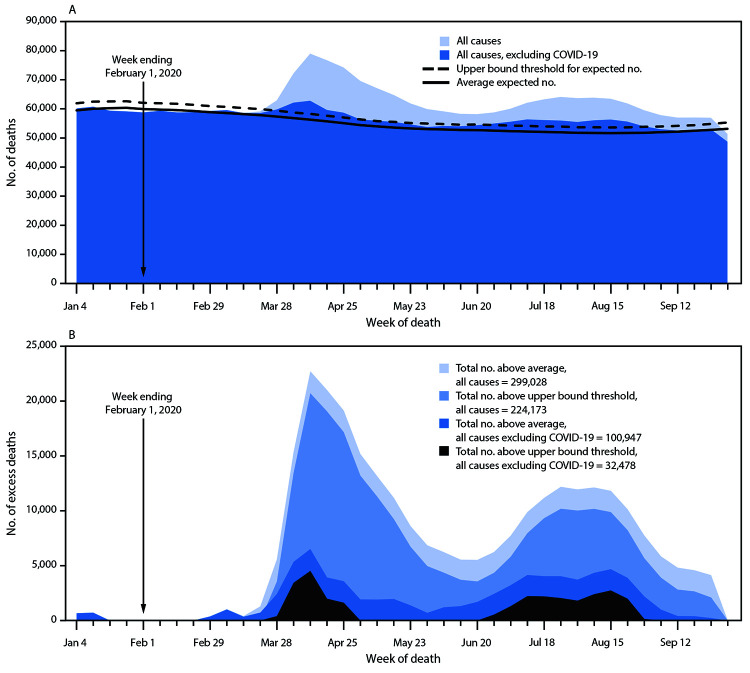
Weekly numbers of deaths from all causes and from all causes excluding COVID-19 relative to the average expected number and the upper bound of the 95% prediction interval (A), and the weekly and total numbers of deaths from all causes and from all causes excluding COVID-19 above the average expected number and the upper bound of the 95% prediction interval (B) — National Vital Statistics System, United States, January–September 2020 **Abbreviation:** COVID-19 = coronavirus disease 2019.

The total number of excess deaths (deaths above average levels) from January 26 through October 3 ranged from a low of approximately 841 in the youngest age group (<25 years) to a high of 94,646 among adults aged 75–84 years.^§§^ However, the average percentage change in deaths over this period compared with previous years was largest for adults aged 25–44 years (26.5%) (Figure 2). Overall, numbers of deaths among persons aged <25 years were 2.0% below average,^¶¶^ and among adults aged 45–64, 65–74 years, 75–84, and ≥85 years were 14.4%, 24.1%, 21.5%, and 14.7% above average, respectively.

**FIGURE 2:**
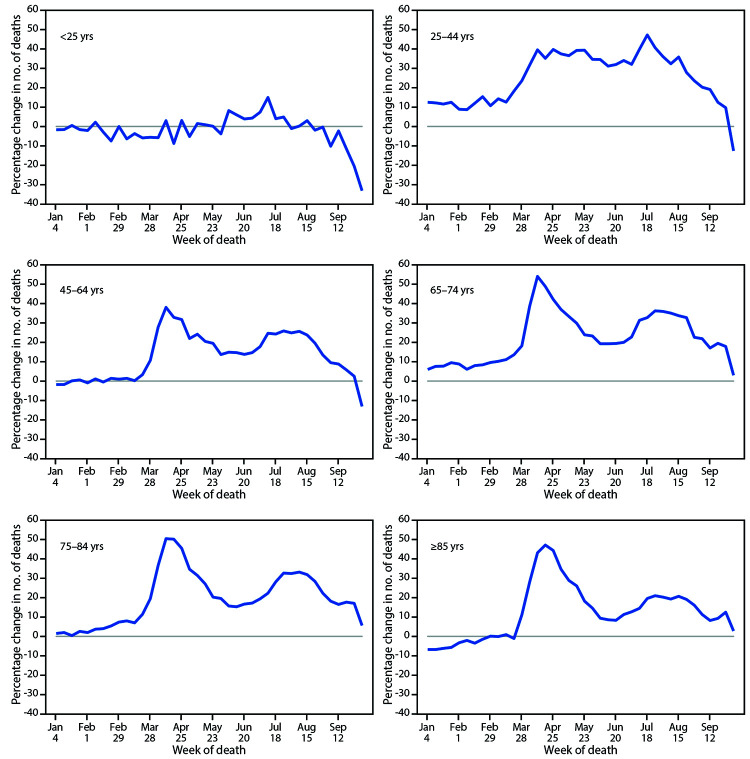
Percentage change in the weekly number of deaths in 2020 relative to average numbers in the same weeks during 2015–2019, by age group — United States, 2015–2019 and 2020

When examined by race and ethnicity, the total numbers of excess deaths during the analysis period ranged from a low of approximately 3,412 among AI/AN persons to a high of 171,491 among White persons. For White persons, deaths were 11.9% higher when compared to average numbers during 2015–2019. However, some racial and ethnic subgroups experienced disproportionately higher percentage increases in deaths (Figure 3). Specifically, the average percentage increase over this period was largest for Hispanic persons (53.6%). Deaths were 28.9% above average for AI/AN persons, 32.9% above average for Black persons, 34.6% above average for those of other or unknown race or ethnicity, and 36.6% above average for Asian persons.

**FIGURE 3:**
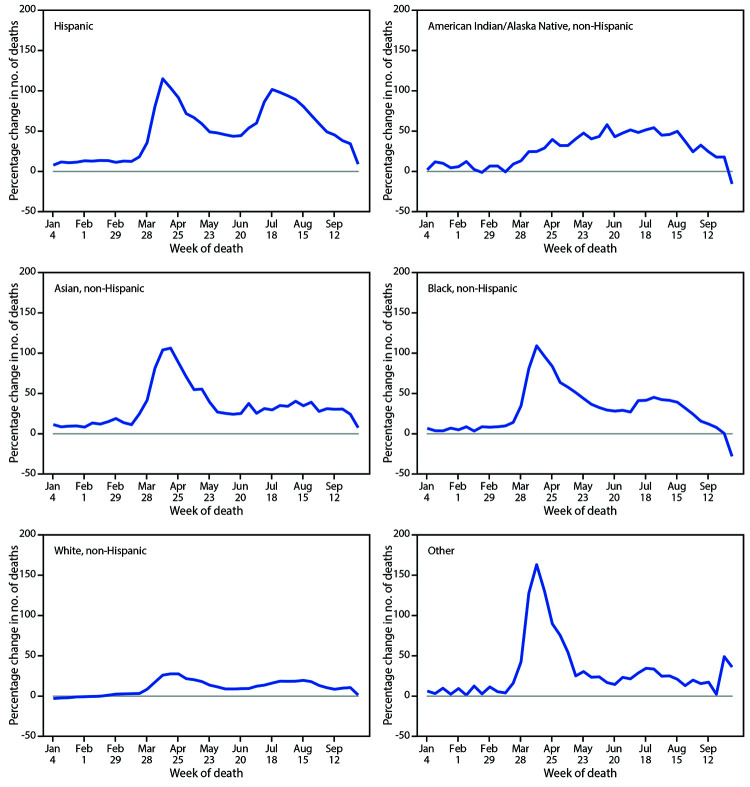
Percentage change in the weekly number of deaths in 2020 relative to average numbers in the same weeks during 2015–2019, by race and Hispanic ethnicity — United States, 2015–2019 and 2020

## Discussion

Based on NVSS data, excess deaths have occurred every week in the United States since March 2020. An estimated 299,028 more persons than expected have died since January 26, 2020; approximately two thirds of these deaths were attributed to COVID-19. A recent analysis of excess deaths from March through July reported very similar findings, but that study did not include more recent data through September (*5*).

Although more excess deaths have occurred among older age groups, relative to past years, adults aged 25–44 years have experienced the largest average percentage increase in the number of deaths from all causes from late January through October 3, 2020. The age distribution of COVID-19 deaths shifted toward younger age groups from May through August (*9*); however, these disproportionate increases might also be related to underlying trends in other causes of death. Future analyses might shed light on the extent to which increases among younger age groups are driven by COVID-19 or by other causes of death. Among racial and ethnic groups, the smallest average percentage increase in numbers of deaths compared with previous years occurred among White persons (11.9%) and the largest for Hispanic persons (53.6%), with intermediate increases (28.9%–36.6%) among AI/AN, Black, and Asian persons. These disproportionate increases among certain racial and ethnic groups are consistent with noted disparities in COVID-19 mortality.***

The findings in this report are subject to at least five limitations. First, the weighting of provisional NVSS mortality data might not fully account for reporting lags, particularly in recent weeks. Estimated numbers of deaths in the most recent weeks are likely underestimated and will increase as more data become available. Second, there is uncertainty associated with the models used to generate the expected numbers of deaths in a given week. A range of values for excess death estimates is provided elsewhere (*7*), but these ranges might not reflect all of the sources of uncertainty, such as the completeness of provisional data. Third, different methods or models for estimating the expected numbers of deaths might lead to different results. Estimates of the number or percentage of deaths above average levels by race/ethnicity and age reported here might not sum to the total numbers of excess deaths reported elsewhere, which might have been estimated using different methodologies. Fourth, using the average numbers of deaths from past years might underestimate the total expected numbers because of population growth or aging, or because of increasing trends in certain causes such as drug overdose mortality. Finally, estimates of excess deaths attributed to COVID-19 might underestimate the actual number directly attributable to COVID-19, because deaths from other causes might represent misclassified COVID-19–related deaths or deaths indirectly caused by the pandemic. Specifically, deaths from circulatory diseases, Alzheimer disease and dementia, and respiratory diseases have increased in 2020 relative to past years (*7*), and it is unclear to what extent these represent misclassified COVID-19 deaths or deaths indirectly related to the pandemic (e.g., because of disruptions in health care access or utilization).

Despite these limitations, however, this report demonstrates important trends and demographic patterns in excess deaths that occurred during the COVID-19 pandemic. These results provide more information about deaths during the COVID-19 pandemic and inform public health messaging and mitigation efforts focused on the prevention of infection and mortality directly or indirectly associated with the COVID-19 pandemic and the elimination of health inequities. CDC continues to recommend the use of masks, frequent handwashing, and maintenance of social distancing to prevent COVID-19.^†††^

**Summary**

**What is already known about this topic?**

As of October 15, 216,025 deaths from COVID-19 have been reported in the United States; however, this might underestimate the total impact of the pandemic on mortality.

**What is added by this report?**

Overall, an estimated 299,028 excess deaths occurred from late January through October 3, 2020, with 198,081 (66%) excess deaths attributed to COVID-19. The largest percentage increases were seen among adults aged 25–44 years and among Hispanic or Latino persons.

**What are the implications for public health practice?**

These results inform efforts to prevent mortality directly or indirectly associated with the COVID-19 pandemic, such as efforts to minimize disruptions to health care.

## Footnotes

* CDC official counts of cases and deaths are released daily at https://covid.cdc.gov/covid-data-tracker/.

† https://www.medrxiv.org/content/10.1101/2020.06.06.20120857v1.full.pdf.

§
https://www.cdc.gov/nchs/nvss/deaths.htm .

¶ Deaths from all causes excluding COVID-19 are calculated by subtracting the number of confirmed or presumed COVID-19 deaths from the total number of deaths. Deaths with confirmed or presumed COVID-19 are assigned the *International Classification of Diseases, Tenth Revision* code U07.1 as a contributing or underlying cause of death on the death certificate.

** Excess deaths over this period ranged from 224,173 to 299,028. The lower end of this range corresponds to the total number above the upper bound of the 95% prediction intervals, and the upper end of the range corresponds to the total number above the average expected counts. Deaths above the upper bound threshold are significantly higher than expected. https://www.cdc.gov/nchs/nvss/vsrr/covid19/excess_deaths.htm.

†† Excess deaths attributed to COVID-19 were calculated by subtracting the number of excess deaths from all causes excluding COVID-19 from the total number of excess deaths from all causes. These excess death estimates were based on the numbers of deaths above the average expected number. Using the upper bound of the 95% prediction interval for the expected numbers (the upper bound threshold), an estimated 224,173 excess deaths occurred during this period, 85.5% of which were attributed to COVID-19.

§§ Weeks when the observed numbers of deaths were below the average numbers from 2015 to 2019 were excluded from the total numbers of excess deaths above average levels (i.e., negative values were treated as 0 excess deaths).

¶¶ The total average percentage change in the number of deaths occurring from the week ending February 1, 2020, through October 3, 2020, included weeks where the percentage difference was negative (i.e., deaths were fewer than expected). This mainly affected the youngest age group, among whom, overall, deaths during this period were 2.0% below average. Excluding weeks with negative numbers of excess deaths results in overall percentage increases of 4.2% for decedents aged <25 years. Increases for other age groups were similar when excluding weeks with negative numbers of excess deaths, with the exception of those aged ≥85 years, among whom the percentage increase was larger (18.1%) when weeks with negative values were excluded.

***
https://www.cdc.gov/coronavirus/2019-ncov/community/health-equity/race-ethnicity.html .

†††
https://www.cdc.gov/coronavirus/2019-ncov/prevent-getting-sick/prevention.html .
